# Oviduct epithelium interactions: roles in sperm selection and embryo quality

**DOI:** 10.1590/1984-3143-AR2025-0035

**Published:** 2025-08-14

**Authors:** Marie Saint-Dizier, Joanna Maria Gonçalves Souza-Fabjan, Karine Reynaud, Pascal Mermillod, Carmen Almiñana, Stefan Bauersachs, Coline Mahé

**Affiliations:** 1 Institut National de Recherche pour l’agriculture, l’alimentation et l’environnement – INRAE, Centre National de la Recherche Scientifique – CNRS, Université de Tours, Physiologie de la Reproduction et des Comportements, Nouzilly, France; 2 Faculdade de Medicina Veterinária, Universidade Federal Fluminense – UFF, Niterói, RJ, Brasil; 3 Department of Reproductive Endocrinology, University Hospital Zurich, Zurich, Switzerland; 4 Institute of Veterinary Anatomy, Vetsuisse Faculty, University of Zurich, Lindau, Switzerland

**Keywords:** oviduct, fallopian tube, embryo, gamete, spermatozoa

## Abstract

This review provides an up-to-date overview of the roles of the oviduct during the periconception period and underlying mechanisms. The functions of the oviduct before, during, and after fertilization are highlighted, with special focus on the effects of epithelial cell contact and luminal secretions on sperm selection mechanisms and acquisition of fertilization ability. The current knowledge on how the oviduct contributes to support fertilization and embryo development via the overall physical milieu (oxygen tension, fluid current, ciliated epithelial cells) and the role of its secretions is also provided. Altogether, the review underlines the unique role of the oviduct during gamete selection and early embryo development, which so far has not been completely possible to mirror when assisted reproductive technologies (ART) are used. Unveiling the most important functional components of oviductal secretions that contribute to better sperm selection, and boost sperm fertilizing ability and early embryo development, can indeed be useful to improve the outcomes of current *in vitro* systems used in ART.

## Introduction

**Figure 1:**
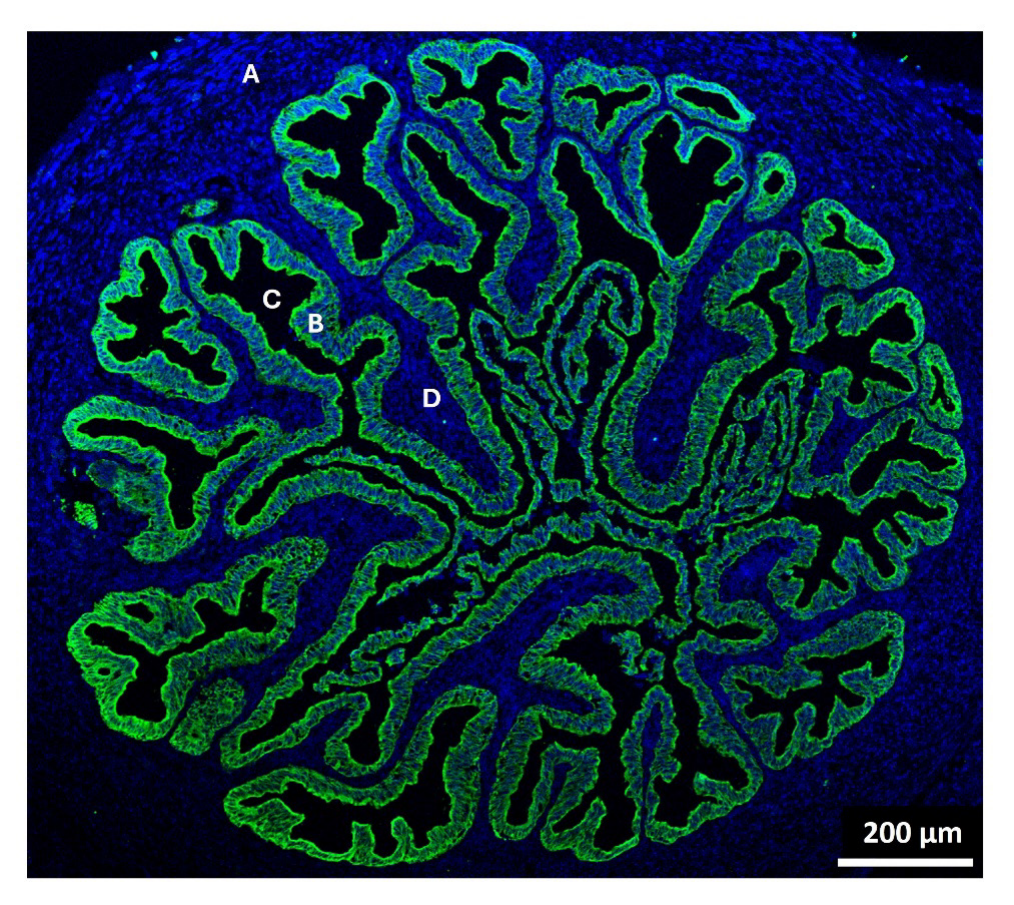
Cross-section of a bovine ampulla at the pre-ovulatory stage of cycle. Green = pan cytokeratin (epithelial cell marker); blue = nuclei (Hoechst staining). (A) smooth muscle; (B) epithelium; (C) lumen; (D) stroma.

The oviduct is one of the least accessible organs of the female body, located deep in the abdomen and except in primates, partly enclosed in an ovarian bursa, with a very small diameter (< 3-5 mm) and a lumen with a labyrinth of mucosa folds (Yaniz et al., 2000) (Figure 1). This complex anatomy, combined with the low number of gametes and embryos transiting within its lumen, poses considerable challenges to observe gamete/embryo-oviduct interactions with current live *in vivo* imaging techniques. *In situ* pictures of spermatozoa and embryos were recently obtained in living mice, but particularly from the ampulla and with limited resolution (Wang and Larina, 2018, 2021). Thus, most data reported so far on oviduct interactions with gametes and embryos, and gathered in this review, have been obtained using *in vitro* models of the oviduct epithelium, including monolayers of oviduct epithelial cells (OECs), oviduct explants or aggregates, and oviduct spheroids.

Within the oviduct, key reproductive processes take place: sperm and egg transport, final sperm maturation, oocyte nuclear maturation (in dogs) and zona pellucida hardening, fertilization, and the first embryonic divisions. Gamete maturation, fertilization, and embryonic development until the blastocyst stage can also be achieved *in vitro* when assisted reproductive technologies (ART) are used, resulting in the birth of healthy offspring. However, bypassing the oviduct might come with a price, leading to fewer selection steps for spermatozoa and poor-quality embryos. In cattle, for example, over the past 25 years, the pregnancy rates of recipient cows carrying *in vitro*-produced (IVP) embryos have been 10 to 40% lower than with *in vivo*-derived embryos, and only 27% of cows receiving IVP embryos delivered a living calf (Ealy et al., 2019). The aim of this review is to provide an up-to-date overview of the role of the oviduct milieu on sperm selection and embryo quality, but also to highlight the benefits of using different *in vitro* oviduct models to understand the underlying mechanisms and mimic the gamete/embryo–oviductal interactions before, during, and after fertilization.

## Before fertilization

Before entering the female genital tract, a significant remodeling of the sperm surface operates in the epididymis and during ejaculation by mixing with the seminal plasma. Spermatozoa at ejaculation are not able to fertilize and acquire this capacity, named capacitation, in the female genital tract: in that end, previous interactions in the male tract have a crucial role in the sperm selection described below and fertility (Rodriguez-Martinez et al., 2024). Although beyond the scope of this review, the seminal plasma also interacts with the female tract and triggers cellular and molecular changes in its luminal epithelium, which contribute to sperm selection and the fertilization success (for reviews, see (Robertson, 2007; Bromfield, 2014).

### The utero-tubal junction selects a subpopulation of sperm entering the oviduct

**Figure 2:**
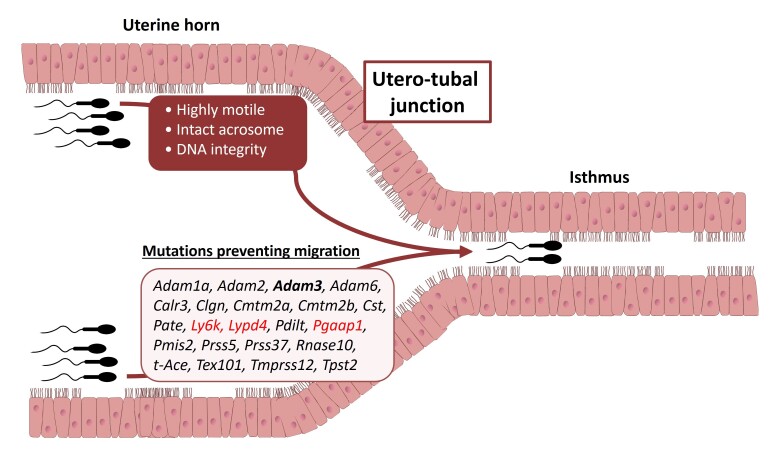
Morphological and molecular factors for sperm passage through the utero-tubal junction. In mice, only highly motile sperm with an intact acrosome can pass the utero-tubal junction (Hourcade et al., 2010; Muro et al., 2016). Sperm from mice deficient for 22 genes, including 19 involved in Adam3 expression (in black) and 3 Adam3-independent ones (in red), were not able to cross the utero-tubal junction (Fujihara et al., 2018, 2019; Larasati et al., 2020; Xiong et al., 2019), suggesting their involvement in sperm migration toward the oviduct.

When artificial insemination (AI) is performed in the cervix, like in sows and sheep, a drastic sperm selection take place in the tract as approximately one out of 10 million sperm reaches the oviduct (First et al., 1968; Hawk et al., 1978). In cows inseminated with millions to billions of sperm into the uterine body, only few hundreds of spermatozoa were counted in the oviducts within 5 to 8 h after AI (Hawk, 1987). The selection rate of motile sperm before IVF in cattle is on average much less restrictive, with approximately 10 to 40% sperm recovery rate after processing with the usual density gradient centrifugation or swim-up (Cesari et al., 2006; Vega-Hidalgo et al., 2022). The very tight utero-tubal junction (UTJ) has dead-end mucosa folds (Yániz et al., 2000) and mucin-rich secretions (Rickard et al., 2019), which constitute a physical barrier for sperm, allowing only the highly motile and morphologically normal ones with an intact acrosome to reach the oviduct (Sostaric et al., 2008; Hourcade et al., 2010; Muro et al., 2016). The UTJ may select sperm according to additional criteria, since a much higher proportion of sperm with no DNA damage was found in the oviduct compared to the uterine cavity of mice after mating (Hourcade et al., 2010). Furthermore, 22 genes, including those coding for ADAM metallopeptidase domain 3 (ADAM3), a sperm surface protein, were identified as essential for sperm passage through the UTJ in mice (Fujihara et al., 2018, 2019; Xiong et al., 2019; Larasati et al., 2020) (Figure 2). Male mice deficient for each of these genes were sterile despite normal sperm morphology and motility, highlighting the crucial role of molecular interactions in sperm migration toward the oviduct. However, in non-rodent mammals, the existence of such a sperm molecular passport is still not known.

Sperm transport in the oviduct is believed to be achieved through the combined effects of muscular contractions, OEC cilia beating, and the luminal fluid microflows (Ezzati et al., 2014), however, the relative contribution of these three mechanisms remains unclear. Recent data in a knockout mouse model lacking motile cilia evidenced that ciliary beating facilitates but is not mandatory for sperm transport toward the ampulla (Yuan et al., 2021). Yet, given the anatomical particularities of the oviduct in mice, the presence of motile cilia may be crucial for sperm transport and final maturation in other mammals (see below).

### A sperm reservoir forms thanks to specific molecular interactions within the isthmus

**Figure 3:**
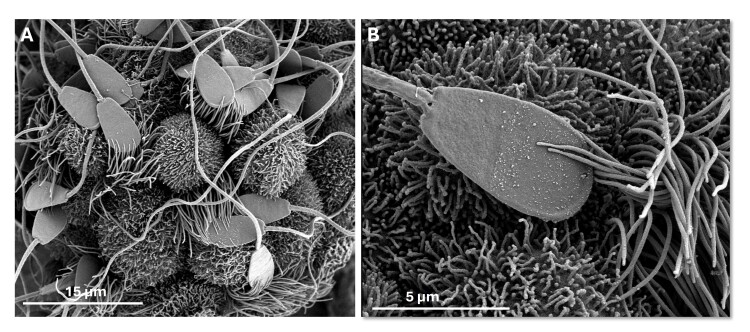
Bull sperm bound to motile cilia of the oviduct epithelium. (A) Scanning electron microscopy picture obtained after co-incubation of frozen-thawed density gradient-washed bull sperm with isthmic epithelial spheroids in a non-capacitating medium; (B) Higher magnification showing a sperm bound with its head to the distal extremity of motile cilia. Microvilli at the surface of secretory non-ciliated cells are seen around the sperm head. Note that all spermatozoa are acrosome-intact with a normal morphology.

Maintaining sperm viable in the oviduct during the pre-ovulatory period is crucial due to the considerable variation in the timing between the onset of estrus and ovulation in most mammalian females (Kemp and Soede, 1997; Saumande and Humblot, 2005). From a practical point of view, increasing sperm viability would allow for a decrease in the number of inseminations needed per pregnancy and the time-consuming detection of estrus (Kemp and Soede, 1997; Soede and Kemp, 1997). One key interaction with the oviduct epithelium for sperm lifespan takes place in the distal segment of the isthmus, where sperm may survive for hours to days, or even months in some bat species (Holt and Fazeli, 2016). For example, in heifers mated at the beginning of estrus, sperm can be held as long as 18-24 h in the caudal isthmus (Wilmut and Hunter, 1984; Hawk, 1987). In gilts mated early in estrus, sperm may survive for 36 h or more in the caudal isthmus (Hunter, 1984). Beyond sperm viability, the sperm reservoir is believed to synchronize gamete meeting and decrease the risk of polyspermy by allowing the progressive release of sperm toward the ampulla, where fertilization occurs (Hunter, 2012; Miller, 2018). The mechanisms of sperm binding to OECs are not completely understood but it is well established that only motile acrosome-intact sperm bind by their head to the extremity of tubal cilia, which firmly grip to the sperm pre-acrosomal region (Suarez et al., 1991; Sostaric et al., 2008; Camara Pirez et al., 2020; Mahe et al., 2023b; Schmaltz et al., 2024b) (Figure 3). Live imaging of sperm co-incubated with oviduct mucosa revealed that immotile sperm were unable to attach and rapidly eliminated by the fluid flow generated by ciliary beating while attached sperm had an active tail beating at the time of binding (Camara Pirez et al., 2020).

**Figure 4:**
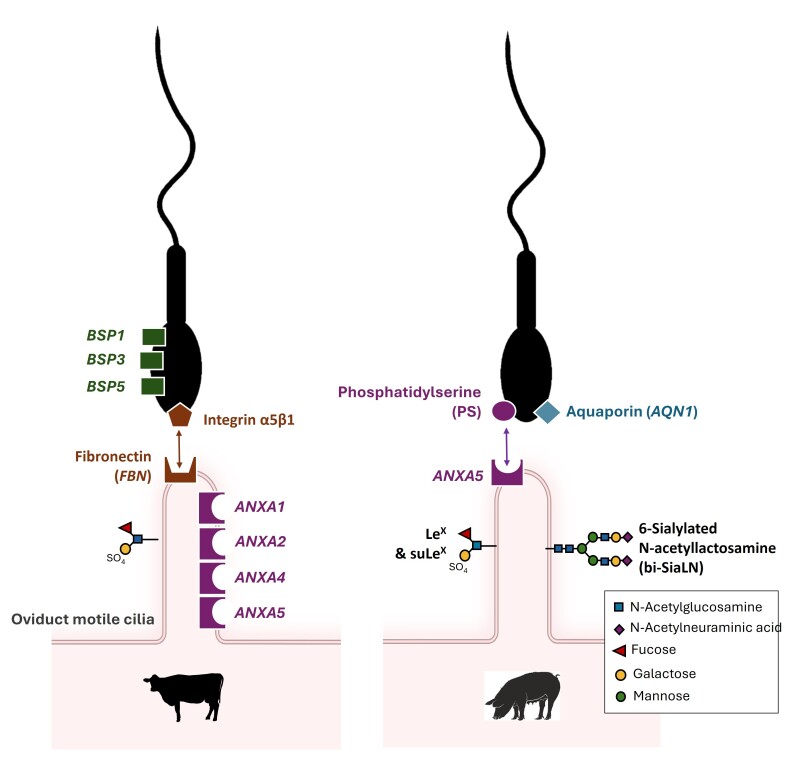
Proteins and phospholipids identified for sperm binding to oviduct epithelial cells Two ligand-receptor couples have been identified so far: the integrin α5β1-fibronectin couple in cattle (brown, on the left; Osycka-Salut et al., 2017) and the phosphatidylserine (PS)-annexin A5 couple in pigs (purple, on the right; Schmaltz et al., 2024a). Proteins from the seminal plasma, including binder of sperm proteins (BSP) 1,3 and 5 in cattle (green, on the left; Gwathmey et al., 2006) and aquaporin (AQN1; Ekhlasi-Hundrieser et al., 2005) in pigs (blue, on the right) have been proposed as additional sperm ligands to cilia. On the female side, several annexins (ANX) were identified as sperm receptors in cattle and pigs (in purple, on cilia) (Ignotz et al., 2007; Teijeiro et al., 2009; Schmaltz et al., 2024a). In addition, boar sperm bind specifically to specific glycans, including Lewis X trisaccharide (Le^X^), 3'-O-sulfated form of Le^X^, and 6-sialylated N-acetyllactosamine (bi-SiaLN), while bull sperm bound 3'-O-sulfated form of Lewis A trisaccharide (Kadirvel et al., 2012; Machado et al., 2014; Dutta et al., 2019a).

Specific glycan motifs that are part of the luminal glycocalyx of OECs (Kadirvel et al., 2012; Machado et al., 2014; Dutta et al., 2019a), and non-glycosylated membrane proteins such as annexins (Ignotz et al., 2007; Teijeiro et al., 2009; Schmaltz et al., 2024a), have been proposed as sperm receptors on oviduct epithelial cells in mammals (for details, see Figure 4). Although most sperm ligands for these oviductal receptors remain to be determined, our group recently showed that phosphatidylserine (PS) exposed on the heads of motile sperm undergoing capacitation interact with annexin A5 on oviduct epithelial cilia (Schmaltz et al., 2024a). Furthermore, the seminal plasma proteins that coat the sperm membrane at ejaculation, including the spermadhesins BSP 1, 3, and 5 in cattle (Gwathmey et al., 2006) and AQN1 in pigs (Ekhlasi-Hundrieser et al., 2005), were found to mediate sperm binding to OECs, possibly through fucose interaction in cattle (Lefebvre et al., 1997).

### Are spermatozoa selected through binding to oviduct epithelial cells?

By using million-per-mL sperm concentrations co-cultured with OEC monolayers for 30 to 60 min, the reported proportions of bound sperm varied from 20-30% for bulls (Gualtieri and Talevi, 2003) to 50-60% for boars (Lopez-Ubeda et al., 2017), including only motile and morphologically normal sperm with an intact acrosome (Gualtieri and Talevi, 2003; Lamy et al., 2017; Lopez-Ubeda et al., 2017; Camara Pirez et al., 2020). These studies compared bound sperm and those that remained unbound in the co-culture medium in order to evaluate whether sperm binding is a selective process. Although for this purpose, supraphysiological sperm concentration, e.g. above mentioned has been used, which probably induces a saturation of sperm binding sites on OECs. Compared to the unbound population, bound sperm displayed higher membrane and DNA integrity in humans (Ellington et al., 1999), horses (Leemans et al., 2014), and cattle (Kon et al., 2009; Nag et al., 2021), in accord with the above hypothesis. Furthermore, the density of bound sperm per oviduct explant surface has been positively associated with conception rates after AI for bulls (De Pauw et al., 2002; Saraf et al., 2019; Nag et al., 2021; Donnellan et al., 2022) and boars (Waberski et al., 2006; Daigneault et al., 2015; Winters et al., 2018). Although a high variability among ejaculates of individual bulls and boars was reported (Camara Pirez et al., 2020; Donnellan et al., 2022; Schmaltz et al., 2024b), using sperm ability to bind to OECs has been proposed to predict male fertility in complement to traditional quality assessment methods (Daigneault et al., 2015; Winters et al., 2018).

In the above studies, a proportion of unbound spermatozoa would probably be able to bind with more explant or spheroid surface available and could not be considered as “low-quality” spermatozoa. *In vivo,* it is still not known if sperm-cilia interaction is a prerequisite for fertilization. However, given the length of the oviduct (5-30 cm among mammals), the proportion of multi-ciliated cells lining its tight lumen (around 25% at estrus (Ito et al., 2016)), and the low number of sperm entering its lumen (a few thousands), it is likely that the large majority, if not all, sperm interact with oviduct cilia during their migration towards oocytes. In accord, bull sperm interact with isthmic and ampullar epithelial cells with similar densities and behaviors *in vitro* (Sostaric et al., 2008; Ardon et al., 2016; El-Sokary et al., 2022). Therefore, there is probably no sperm selection *per se* through binding to oviduct cilia *in vivo*, but rather a variable response of sperm cells to binding, leading to variable ability to survive and undergo capacitation on time.

### Binding to OECs regulates sperm capacitation and oxidative stress

Spermatozoa in the female genital tract mostly rely on their environment to delay or induce capacitation. Delaying sperm capacitation before ovulation may be critical to maintain a subpopulation of viable sperm within the female tract. Capacitation comprises multiple steps, including cholesterol efflux and PS externalization at the membrane, calcium influx, increased tyrosine phosphorylation of proteins, and an asymmetrical flagellar beating, called hyperactivated motility (Puga Molina et al., 2018). Sperm are particularly susceptible to oxidative stress due to very little cytoplasmic content and inadequate cell repair systems (Dutta et al., 2019b). Human sperm bound to oviductal membrane proteins displayed lower intracellular ROS levels (Huang et al., 2013), suggesting that binding to OECs may protect sperm against oxidative stress. Similarly, binding of boar sperm to their oviductal glycan ligand (suLeX) decreased their production of intracellular ubiquinone, fumarate (one component of the citric acid cycle) and ROS (Hughes et al., 2023), suggesting that binding to specific oviduct glycans triggers a reduction in mitochondrial activity that delays capacitation and lengthens sperm lifespan. However, the reported effects of sperm binding to OECs on capacitation are not consistent. Boar sperm binding to OECs or oviduct glycans was reported to inhibit calcium influx (Machado et al., 2020), PS externalization (Lopez-Ubeda et al., 2017), and protein tyrosine phosphorylation (Luno et al., 2013; Lopez-Ubeda et al., 2017). On the contrary, some studies reported a stimulation of the cyclic AMP/protein kinase A pathway leading to acrosome reaction in human (Martinez-Leon et al., 2015) and bull sperm (Osycka-Salut et al., 2020) after binding to fibronectin, a glycoprotein present on the luminal surface of oviduct ciliated cells (Makrigiannakis et al., 2009; Osycka-Salut et al., 2017). The binding to oviduct explants was also shown to induce protein tyrosine phosphorylation in stallion (Leemans et al., 2014) and boar (Petrunkina et al., 2004) spermatozoa. A similar positive effect on capacitation was observed after sperm exposure to peri-ovulatory oviduct fluid (OF) in a number of species, including cattle (Bergqvist et al., 2006; Kumaresan et al., 2019), pigs (Kumaresan et al., 2012), and sheep (El-Shahat et al., 2018). Of note, when using oviduct explants, a combined effect of sperm-to-cilia contact and OECs secretions in the vicinity of bound sperm is observed, which may contribute to the variability in sperm response according to female cell physiology. Further studies are needed to assess whether the introduction of standardized batches of oviductal compounds (sperm ciliary ligand, frozen or lyophilized oviduct fluid) into standard IVF protocols could improve their outcomes.

### Oviduct secretions deliver key molecules for sperm maturation

The oviduct epithelium releases soluble ions and molecules which can interact directly with gametes/embryos, and molecules enclosed in luminal extracellular vesicles (EVs), also called oviductosomes (Saint-Dizier et al., 2019). Oviduct EVs include small (40-100 nm) and larger vesicles (100-1000 nm) and have been shown to interact with the sperm membrane in a number of species, including pigs (Alcântara-Neto et al., 2020a; Toledo-Guardiola et al., 2024), cattle (Franchi et al., 2020), horses (Lange-Consiglio et al., 2022), cats (Ferraz et al., 2019), and mice (Al-Dossary et al., 2013; Al-Dossary et al., 2015; Bathala et al., 2018). Evaluation of the functional impact of these interactions showed that oviduct EVs modulate sperm motility (Ferraz et al., 2019; Alcântara-Neto et al., 2020a), survival (Alcântara-Neto et al., 2019), processes of capacitation such as protein kinase A phosphorylation (Toledo-Guardiola et al., 2024), protein tyrosine phosphorylation and calcium influx (Franchi et al., 2020), acrosome reaction (Ferraz et al., 2019; Franchi et al., 2020; Lange-Consiglio et al., 2022), and the ability to fertilize oocytes *in vitro* (Ferraz et al., 2019; Lange-Consiglio et al., 2022; Toledo-Guardiola et al., 2024).

Some crucial molecules for conception may be delivered to sperm by secretions of the female reproductive tract. One example is sperm adhesion molecule 1 (SPAM1, also named PH-20), a highly conserved hyaluronidase GPI-linked to the sperm membrane that plays roles in penetration through cumulus cells, adhesion to the zona pellucida, and acrosome reaction (Martin-DeLeon, 2006). Although the initial acquisition of SPAM1 takes place in the epididymis through fusion with epididymosomes, sperm are also exposed to SPAM1 in the uterine and oviduct fluids at estrus, as shown in mice (Zhang and Martin-DeLeon, 2003; Griffiths et al., 2008b). Sperm exposed to estrous uterine fluid take up SPAM1 over the acrosome and midpiece of the flagella, leading to an enhanced ability to bind hyaluronic acid (Griffiths et al., 2008a, b).

Another example is the membrane protein calcium ATPase 4 (PMCA4), a major calcium pump, whose deletion leads to a loss of sperm motility and male infertility in mice (Al-Dossary et al., 2013). PMCA4 is highly expressed in the oviduct, and its concentration in the OF is up to 9-fold higher than in other parts of the female reproductive tract in mice at estrus (Al-Dossary et al., 2013). Transmission immunoelectron and high-resolution structured illumination microscopy have evidenced the delivery of PMCA4 to the sperm head and the flagellum midpiece membrane via fusion of oviduct EVs involving integrins and CD9 (Al-Dossary et al., 2013, 2015). The delivery of calcium pumps to sperm via female secretions remains to be explored in other mammals, but the presence of PMCA1 and PMCA4 in human OF EVs (Bathala et al., 2018) suggests that it might be a conserved process.

## During fertilization

### Spermatozoa pre-bound to oviduct epithelial cells display higher fertilizing ability

**Figure 5:**
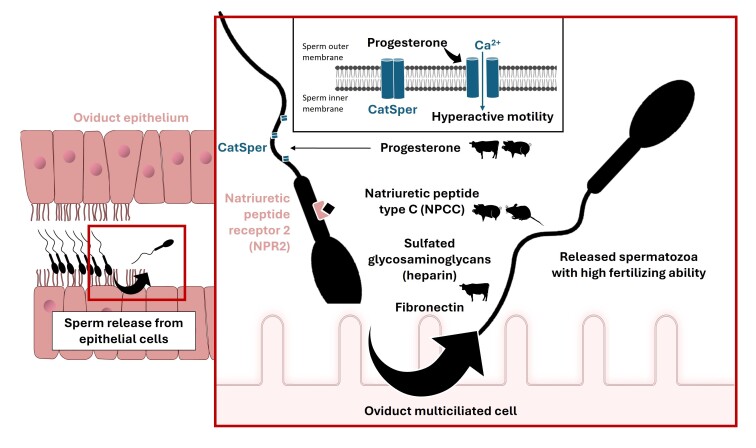
Molecular signals inducing sperm release from oviduct epithelial cells. Progesterone in cattle (Lamy et al., 2017; Romero-Aguirregomezcorta et al., 2019) and pigs (Machado et al., 2019); natriuretic peptide type C (NPCC) in mice (Wang et al., 2022) and pigs (Wu et al., 2023); sulfated glycosaminoglycans in cattle (Mahe et al., 2023b; Talevi and Gualtieri, 2001); fibronectin in cattle (Osycka-Salut et al., 2017, 2020).

The timed release of spermatozoa from the caudal isthmus towards the ampulla, where cumulus-oocyte complexes progress after ovulation, is a prerequisite for fertilization. The exact mechanisms leading to sperm release are not fully understood but they likely involve endocrine and probably paracrine signals in the oviduct epithelium. Following the luteinization of pre-ovulatory follicles, levels of progesterone dramatically rise in the OF, reaching around 30 nM just after ovulation in sows and cows (Ballester et al., 2014; Lamy et al., 2016b). Nanomolar concentrations of progesterone have been shown to induce a CatSper-mediated hyperactivated motility and release of bound sperm from OECs in cattle and pigs (Lamy et al., 2017; Machado et al., 2019; Romero-Aguirregomezcorta et al., 2019). Other compounds fluctuating in concentrations in the OF around ovulation time, including fibronectin (Osycka-Salut et al., 2017, 2020) and heparin-like sulfated glycosaminoglycans (sGAG) (Talevi and Gualtieri, 2001; Bergqvist and Rodriguez-Martinez, 2006; Mahe et al., 2023b), may act synergistically with progesterone on sperm release. Sperm release may be also facilitated by natriuretic peptide type C (NPCC) expressed in ampullar epithelial cells (Wang et al., 2022; Wu et al., 2023). In response to the contact with mature cumulus-oocyte complexes, NPCC expression was enhanced in porcine and mouse ampulla, while its receptor (NPR2) was found on the midpiece of sperm (Wang et al., 2022; Wu et al., 2023) (Figure 5). Nanomolar concentrations of NPCC promoted pig sperm release from isthmic explants mechanism implying calcium and cGMP-sensitive cyclic nucleotide-gated channels (Wu et al., 2023).

Examination of the sperm subpopulation released from OECs by the action of fibronectin showed higher progressive motility with signs of capacitation (Osycka-Salut et al., 2017). A practical limitation of using sperm pre-bound to OEC for *in vitro* fertilization (IVF) is the limited numbers of sperm that can be recovered (less than 50% of the bound population, i.e. < 25% of the initial population) and their quantification before IVF, which requires extra manipulation. Another limitation is the use of frozen-thawed semen as the freezing process induces a destabilization of the sperm membrane and capacitation-like changes, which were reported to decrease the ability of sperm to bind to OECs *in vitro* in cattle (Goldman et al., 1998) and pigs (Tomas et al., 2013). Nevertheless, studies that used sperm pre-bound to OEC then released for IVF evidenced an increase in the rates of oocyte penetration and cleavage compared to unbound or not selected control sperm, using frozen-thawed semen in cattle (Gualtieri and Talevi, 2003; Kon et al., 2009; Lamy et al., 2017; El-Sokary et al., 2022) and fresh semen in pigs (Bureau et al., 2000; Lopez-Ubeda et al., 2017) (see Table 1 for details). Higher cleavage rates were observed when bull sperm were pre-bound to explants from the isthmus compared to ampulla or infundibulum (El-Sokary et al., 2022), suggesting a region-specific effect of binding-release on sperm fertilizing ability. Furthermore, the use of boar sperm pre-bound to porcine OECs (Bureau et al., 2000) or to suLeX glycan motifs (Soto-Heras et al., 2025) increased the numbers of monospermic zygotes after IVF. Our group also examined the blastocyst rate, which was enhanced after IVFwhen sperm was pre-bound to OECs and released by progesterone compared to controls in cattle (Lamy et al., 2017). Overall, the enhanced fertilizing ability was obtained after only 30 to 60 min of sperm binding to OECs (Gualtieri and Talevi, 2003; Lamy et al., 2017; Soto-Heras et al., 2025).

**Table 1:** Studies reporting an improvement in the *in vitro* fertilization ability of sperm after binding to oviductal epithelial cells or glycans and release.

**Species**	***In vitro* oviduct model**	**Sperm preparation**	**Main result**	**Reference**
Cattle	OEC monolayer from the whole oviduct of pubertal cows	Frozen-thawed and Percoll-gradient washed bull semen	Sperm bound to OEC and then released by heparin displayed higher ZP binding capacity and produced higher cleavage rates after IVF, compared with unbound and control sperm.	Gualtieri and Talevi (2003)
Cattle	OEC suspension from the whole oviduct of pubertal cows at post-ovulatory stage	Frozen-thawed and Percoll-gradient washed bull semen	Unbound free sperm displayed lower ZP binding capacity and produced less fertilized COCs or denuded oocytes, compared with a mix of bound and unbound sperm (control).	Kon et al. (2009)
Cattle	OEC monolayer from the whole oviduct of pubertal cows at peri-ovulatory stage	Frozen-thawed and Percoll-gradient washed bull semen	Sperm bound to OEC and then released by P4 produced higher cleavage and blastocyst rates after IVF, compared with controls without OEC	Lamy et al. (2017)
Cattle	Cell aggregates from isthmus of pubertal cows	Frozen-thawed bull semen pre-selected by swim up	Sperm bound to aggregates from isthmus produced higher cleavage rates on day 2 after IVF, compared with control sperm.	El-Sokary et al. (2022)
Pig	OEC monolayer	Fresh Percoll-gradient washed boar semen	Sperm pre-incubated with OEC produced higher rates of zygotes with two pronuclei and reduced polyspermy compared with controls without cells.	Bureau et al. (2000)
Pig	OEC monolayer from the whole oviduct of cycling gilts	Fresh Percoll-gradient washed boar semen	Sperm bound to OEC and then co-incubated with oocytes produced higher penetration rates and nuclear decondensation after IVF, compared with unbound sperm.	Lopez-Ubeda et al. (2017)
Pig	Oviduct glycans coupled to a glass surface	Fresh Percoll-gradient washed boar semen	Sperm bound to sulfated Lewis X trisaccharide and then released by COCs produced higher rates of monospermic zygotes, compared with control sperm with no pre-binding.	Soto-Heras et al. (2025)

OEC: oviduct epithelial cells; ZP: zona pellucida; IVF: in vitro fertilization; COCs: cumulus-oocytes complexes.

Taken together, these data support the hypothesis that sperm binding-release processes along the oviduct improve or accelerate the capacity of sperm to fertilize oocytes, maybe through the delivery of key molecules during binding. It may also be the case that the binding process selects a sperm population of higher quality, although the exact characteristics or molecular passport of the selected sperm remains unknown.

### Oviduct secretions favor monospermic fertilization

Oocyte polyspermy during IVF is a common problem, particularly frequent in pigs and goats, and leading to early embryo demise. Supplementation with OF, OEC secretions, or oviductal proteins during IVF reduced the rate of polyspermic zygotes while maintaining good rates of oocyte penetration, as reported in goats (Bragança et al., 2021), horses (Mugnier et al., 2009; Ambruosi et al., 2013), and pigs (Romar et al., 2001; Batista et al., 2016; Alcântara-Neto et al., 2020b). A similar beneficial effect on polyspermy was obtained when using oviduct EVs derived from the OF (Alcântara-Neto et al., 2020b) or OECs monolayers (Fang et al., 2023), resulting in higher blastocyst rates in pigs (Fang et al., 2023).

The aforementioned effects could be mediated through modifications of both gametes, although oocytes are probably the major players. Exposure of oocytes to OF before IVF reproduced the beneficial effects obtained with OF in pigs (Batista et al., 2016). The OF has been shown to induce the hardening of the oocyte zona pellucida within 30 min or less and this ability was correlated with its ability to induce monospermy during IVF in pigs (Mondejar et al., 2013). The actors of the hardening of the zona pellucida have been partly identified, including: oviductal glycoprotein 1 (OVGP1), lactotransferrin (LTF), members of the HSP and PDI protein families, and heparin-like sulfated GAGs (Coy et al., 2008; Mondejar et al., 2013; Zumoffen et al., 2013). All these proteins are abundantly present in the OF around the ovulation time (Mahe et al., 2022). In addition, OVGP1 and members of the heat shock protein (HSP) and protein disulfite isomerase (PDI) families also interact with spermatozoa in both parts of the oviduct (Mahe et al., 2023a) and may modulate sperm adhesion to the zona pellucida. Lactotransferrin has been shown to interact with both spermatozoa and oocytes, causing a significant inhibition of sperm-zona pellucida interaction in humans (Zumoffen et al., 2013). Besides, many of the proteins found abundantly in the OF and with potential contributions to monospermic fertilization have also been identified in oviductal EVs (Alcântara-Neto et al., 2020b). Oviductal EVs interact with the cumulus cells, zona pellucida, and oocyte, being able to cross the zona pellucida and transferring the protein cargo to the oocyte (Alcântara-Neto et al., 2020b). It has been shown that EVs can deliver OVGP1 into the oocyte, which may be a component of the polyspermy regulatory mechanism (Alcântara-Neto et al., 2020b).

## After fertilization

Evidence from different species showed that embryos produced *in vitro*, either from oocytes matured *in vivo* or *in vitro*, are less competent than those developed *in vivo*. In cattle for example, clear differences in morphology and ultrastructure (Fair et al., 2001; Abe et al., 2002; Rizos et al., 2002a, b), energy metabolism (Gardner, 1998; Khurana and Niemann, 2000; Melo-Sterza and Poehland, 2021), gene expression (Knijn et al., 2005; Smith et al., 2009; Driver et al., 2012; Gad et al., 2012), methylation patterns (Salilew-Wondim et al., 2018), and protein composition (Banliat et al., 2022) have been reported between IVP embryos and their *in vivo* counterparts. Furthermore, the ability of bovine IVP embryos to survive after cryopreservation is lower than that of their *in vivo* counterparts (Rizos et al., 2008; Ferre et al., 2020).

On the other hand, the co-culture of IVP embryos with OECs improves blastocyst formation and enhances their cryotolerance in cattle (Cordova et al., 2014b; Schmaltz-Panneau et al., 2015; García et al., 2017; Pranomphon et al., 2024b) and pigs (Lorenzo et al., 2024), evidencing the beneficial effect of the oviductal secretions and/or physical milieu provided by the oviduct epithelium on embryo quality. This raises the key question of what in the oviductal microenvironment is so crucial for achieving optimal embryo development?

### A short stay in the oviduct shapes further embryo development

Embryos spend only a few days in the oviduct before entering the uterus, from 2 days in pigs to 6 days in horse (Table 2), but with a remarkable impact on their development and quality. In cattle, culture of embryos with OEC monolayers (Cordova et al., 2014a), oviduct epithelial spheroids (Pranomphon et al., 2024a), or in OEC-conditioned media (Senn et al., 2024) for the first 3-4 days was enough to improve blastocyst formation and quality on day 8 post-IVF. The use of OEC monolayers on days 1-4 post-IVF led to higher blastocyst rates than days 4-8 or the entire culture time (Cordova et al., 2014a), suggesting that a short initial priming by oviduct secretions is enough to produce long-lasting beneficial effects on embryo development. This was also observed in other species. In sheep, OEC explants during the first 4 days of development increased the blastocyst rates on day 8 compared to controls (Dashti et al., 2016). In pigs, culturing IVF zygotes during the initial two days of culture with OECs (Lorenzo et al., 2024) or oviduct EVs (Alcântara-Neto et al., 2022) was enough to enhance blastocyst rates on day 7. In the same line, embryo co-culture with isthmic epithelial spheroids for the first 4 days of development improved the blastocyst rates at 7 days, and this was observed under both 5% and 20% oxygen (Pranomphon et al., 2024b). Furthermore, the transcriptomic analysis of blastocysts showed that compared to the massive effect of oviduct spheroids, the effect of co-culture time was much lower (hundreds vs. a dozen of differentially expressed genes), indicating that the presence of OECs beyond the 16-cell stage had little additional impact on the number of modulated genes (Pranomphon et al., 2024b). However, the functional analysis revealed that the impacted pathways were more significant after 7 than 4 days of co-culture, indicating that a longer co-culture time did not change the activated pathways but rather the magnitude of gene expression changes (Pranomphon et al., 2024b).

**Table 2:** Embryonic stage of zygotic genome activation (ZGA), time in the oviduct relative to ovulation, and developmental stage on entering the uterus in mammalian species.

	**Cattle**	**Pig**	**Sheep**	**Goat**	**Horse**	**Rabbit**	**Human**	**Mice**
Onset of major ZGA	8-cell	4-cell	8-16 cell	4-8 cell	8-cell	8-cell	4-cell	2-cell
Duration of stay in the oviduct (days)	3 - 4	≈ 2	3 - 4	4	5-6	2 - 3	3 – 3.5	3 – 3.5
				2-2.5				
Embryo stage on entering the uterus	8-16 cell	4-cell	8-16 cell	12-cell	Morula - blastocyst	Morula-blastocyst	12-16 cell	Blastocyst
References	(Betteridge, 1995; Gad et al., 2012)	(Tománek et al., 1989)	(Crosby et al., 1988; Betteridge, 1995)	(Betteridge, 1995; Deng et al., 2020)	(Betteridge, 1995; Goszczynski et al., 2022)	(Betteridge, 1995; Pacheco-Trigon et al., 2002)	(Betteridge, 1995; Jukam et al., 2017)	(Betteridge, 1995; Jukam et al., 2017)

Day 0: ovulation.

Additionally, the culture of cattle embryos with endometrial epithelial cell-conditioned media on days 4-8 post-IVF was recently reported to increase blastocyst formation (Senn et al., 2024). However, the greatest impact on embryo development and quality was obtained after pre-culture in OEC-conditioned media on days 1-3 (Senn et al., 2024), suggesting that reproducing the sequential exposure to oviductal and then endometrial secretions is optimal for embryo development. Time lapse video evidenced that OEC-conditioned media on days 1-3, but not later exposure to uterine secretions, reduced the time to morula compaction and blastocyst formation (Senn et al., 2024). These results suggest that oviduct secretions accelerate further development. Additionally, exposure to OEC conditioned medium decreased the proportion of apoptotic cells, concomitant with an increase in embryo cell numbers and the expression of genes inhibiting apoptosis in bovine blastocysts (Sidrat et al., 2020). Similarly, exposure to oviduct epithelial spheroids during *in vitro* development inhibited genes known to initiate cell apoptosis, like caspase 8 (*CASP8*), and induced the expression of others with anti-apoptotic functions, like caveolin 1 (*CAV1*) and nuclear protein 1 transcriptional regulator (*NUPR1*) in bovine embryos (Pranomphon et al., 2024b).

### Key aspects of the oviduct milieu: specific compartment functions, dynamic microflows, and low oxygen tension

The early embryo migrates from the ampulla to the isthmus during its early development. During this time, the embryo-maternal interactions are likely to be region-specific. In mice, a recent single-cell RNA sequencing of oviduct cells revealed that the ampulla and isthmus have distinct transcriptomic signatures and fetal origins (Ford et al., 2021), suggesting that the oviduct should rather be considered as two organs with distinct physiological roles. In heifers at day 2.5 after insemination, all embryos recovered were located at the beginning of the isthmus (Rodriguez-Alonso et al., 2019). Furthermore, EVs from the isthmus maintained higher blastocyst survival after vitrification compared to those from ampulla in cattle (Lopera-Vasquez et al., 2017a). In sheep, a higher proportion of expanded and hatched blastocysts, and with a greater number of cells, were obtained with isthmic compared to ampullar explants in co-culture (Dashti et al., 2016). Altogether, these data indicate a greater effect of the isthmus on embryo quality compared to other oviduct compartments.

**Figure 6:**
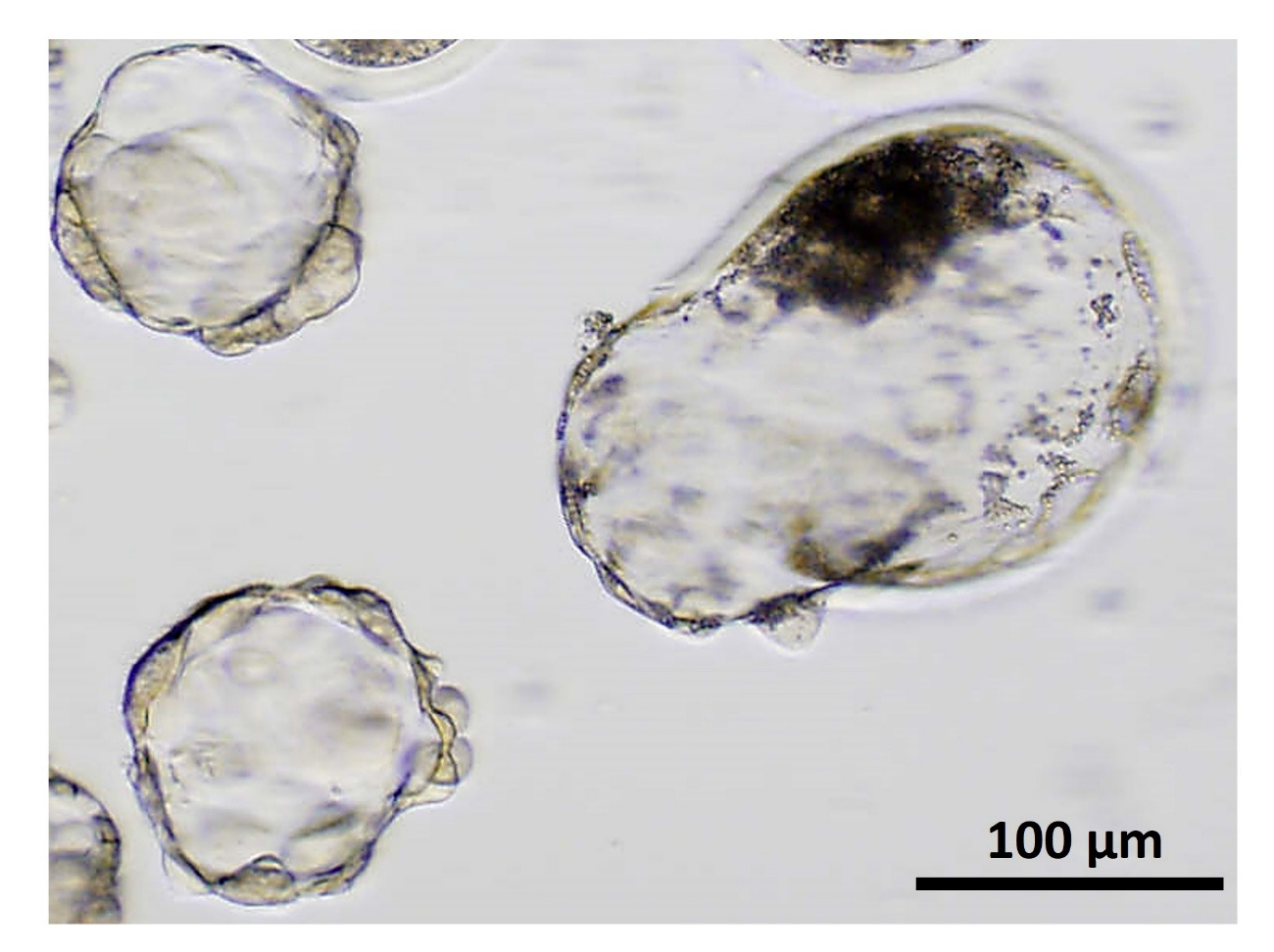
Bright-field picture of a bovine hatching blastocyst (on the right) that developed in co-culture with isthmic epithelial spheroids (two are visible on the left) maintained in suspension in the culture medium thanks to outward ciliary beating.

The oviduct is filled with tubal fluid, which not only nourishes and protects the embryo, but also facilitates the embryo’s transport into the uterus. The volume of fluid of the whole oviduct in the peri-ovulatory period is around 40 µL in cattle and 15 µL in sheep, including less than 5 µL for the isthmus (personal data and (Teteau et al., 2022)). That means that the embryo(s) develop in a semi-fluid microenvironment in contact with the epithelial cells of the mucosa folds (Figure 1). The microfluidic functioning of the oviduct is an important aspect that has been relatively neglected so far. Thanks to the ciliary beating of multiciliated cells and muscular contractions, the tiny volume of luminal secretions is continuously brewed and renewed (Saint-Dizier et al., 2019). Recently, dynamic microfluid culture systems (with average flow rates of 18 nL/min) have been reported to improve the development and quality of bovine, murine, and human embryos compared to static systems (Alegretti et al., 2024). On the contrary, culturing embryos with OF at relatively high concentrations (>2.5%) in a static system had a toxic effect on cattle embryo development (Lopera-Vasquez et al., 2017b), highlighting the importance of medium renewal around the embryos. In this regard, Pranomphon et al. proposed that the beneficial effects on embryo development observed after co-culturing embryos with oviduct spheroids might be due to the capacity of the oviduct spheroids to maintain their outward ciliary beating, moving in suspension, and recreating microflows around embryos (Pranomphon et al., 2024c) (Figure 6).

The oxygen tension in the oviduct lumen has been reported to range between 2 and 8% in various mammalian species (Fischer and Bavister, 1993), and embryos are typically cultured in incubators with oxygen regulation (5% CO_2_ and 5% O_2_). However, OECs typically grow in incubators without oxygen regulation, thus with an oxygen level around 18-20%. The actual needs of OECs are probably intermediate, as the blood capillaries under their basal membrane continuously provide them with oxygen *in vivo*. It has been pointed out that the supporting effect of OECs on embryo development may be associated with their oxygen consumption in the culture medium, reproducing the low oxygen tension in the oviduct lumen. Using incubators without oxygen regulation, blastocyst yields were systematically improved after co-culture with OEC monolayers (Cordova et al., 2014b; Schmaltz-Panneau et al., 2015) or spheroids (Pranomphon et al., 2024b, c). Recent data comparing 5% and 20% oxygen reported very close blastocyst rates, total cell numbers, and gene expression after co-culture with oviduct epithelial spheroids for 7 days (Pranomphon et al., 2024b), indicating that oxygen regulation is not needed if embryos are co-cultured with OECs up to the blastocyst stage. The oxygen consumption may be reproduced with other cells and is not tissue specific. However, although the first cleavages can be supported equally by OECs and other somatic cells such as fibroblasts, a marked improvement in further embryo development and viability was observed with OEC co-culture in sheep (Gandolfi and Moor, 1987). An improvement in blastocyst total cell numbers and gene expression was also observed after co-culture with OEC spheroids compared with controls without cells under both 5% and 20% oxygen (Pranomphon et al., 2024b). These findings indicate that beyond oxygen consumption, the oviduct epithelium exerts through its secretions a specific action on embryo quality.

The oviduct epithelium synthesizes and secretes several antioxidant enzymes into its lumen, including glutathione peroxidase, superoxide dismutase and catalase (Lapointe and Bilodeau, 2003; Schmaltz-Panneau et al., 2015; Lamy et al., 2016a; Mahe et al., 2022), and antioxidant activities were measured in the OF (Lapointe and Bilodeau, 2003). Co-culture of embryos with OECs (Cordova et al., 2014b) or supplementation of the medium with OF (Hamdi et al., 2018) or OEC-derived EVs (Fang et al., 2022) resulted in an increased expression of enzymes involved in ROS scavenging, like glutathione peroxidase 1 (GPX1) or superoxide dismutase 1 (SOD1), in bovine and porcine embryos, with a concomitant decrease in ROS levels in the embryonic cells (Fang et al., 2022; Lorenzo et al., 2024). This suggest that oviduct secretions stimulate the expression of antioxidant pathways in embryonic cells in addition to their probable direct antioxidant activivty. 

### The oviduct milieu modulates the activation of the zygotic genome

A major event in an embryo’s life is the initiation of its own transcriptional program, a process called zygotic genome activation (ZGA). For most mammalian species, the ZGA occurs when embryos are still in the oviduct (Table 2) and developmental arrest is often observed during ZGA in IVP embryos. A comprehensive proteomic analysis of early bovine embryos showed that although *in vivo*- and *in vitro*-derived embryos start their ZGA at the same time, after the 8-cell stage, the increase in proteins involved in RNA metabolism and translation was much slower in *in vivo-*derived embryos but resulted in the same total number of proteins at the blastocyst stage (Banliat et al., 2022). These results were globally in accordance with the “quiet embryo” hypothesis, in which a premature or an excessive activation of the embryonic genome, in response to an unfavorable environment, decreases the ability of an embryo to pursue development (Baumann et al., 2007). Thus, beyond the source of oocytes, known to have a major effect on ZGA (Gad et al., 2012; Dorfeshan et al., 2018), there is evidence that the environment to which zygotes are exposed has also a great impact on their genome reprogramming and resulting blastocysts (Rizos et al., 2002b; Dalvit et al., 2005; Gad et al., 2012; Cordova et al., 2014b; Lonergan and Forde, 2014; Rodríguez-Alonso et al., 2020; Pranomphon et al., 2024b). For instance, exposure to OF during the first 4 days of culture was shown to promote the expression of DNA methyltransferases (DNMT) 1 and 3A in bovine embryos at the 4-cell (Barrera et al., 2017) and blastocyst (Hamdi et al., 2018) stages. The DNA methylation pattern of several blastocyst genes was also altered by the presence of OF (Barrera et al., 2017).

### The oviduct plays an active role in supporting the embryo metabolism

The most crucial nutrients for the early embryo are carbohydrates and amino acids, which provide energy and substrates for protein synthesis and act as players of the epigenetic programming (Milazzotto et al., 2020). Bovine embryos start to consume pyruvate and glucose around the time of ZGA. Then, around the 16-cell to compact morula, they enter the uterus, where concentrations of glucose are higher than in the OF (Hugentobler et al., 2008). At this time, a significant increase in the oxidation of pyruvate, glucose, and lactose has been reported in embryos (Gardner, 1998; Khurana and Niemann, 2000). Transcriptomic studies on bovine embryos evidenced that a majority of differentially expressed genes between *in vivo*- and *in vitro*-derived blastocysts were involved in metabolic processes, including carbohydrate metabolism but also lipid, nucleic acid, and amino acid metabolism (Driver et al., 2012; Gad et al., 2012). Similarly, in porcine embryos, a high proportion of genes related to metabolism was dysregulated when *in vivo*- and *in vitro*-derived blastocysts were compared, most of which were upregulated *in vivo* (Miles et al., 2008). The proteomic analysis of bovine embryos confirmed that *in vivo*-derived embryos, as soon as the 8-12 cell stage, produce higher amounts of key glycolytic enzymes than their *in vitro* counterparts (Banliat et al., 2022), suggesting a more active carbohydrate metabolism when in contact with the oviduct epithelium. In accordance, IVP embryos cultured in OEC-conditioned medium showed four times higher expression of enzymes involved in ATP production, like pyruvate dehydrogenase and glutamate dehydrogenase 1 (GLUD1), compared to controls (Sidrat et al., 2020). The expression of glucose transporters GLUT1 and GLUT5 was also increased in bovine embryos after 8 days of co-culture with OEC (Cordova et al., 2014a).

Altogether, these results indicate that the oviduct epithelium supports embryo growth through an inhibition of cell apoptosis, mitigates oxidative stress through oxygen consumption and specific antioxidant secretions, modulates ZGA, and favors the transition from an oxidative to a glycolytic metabolism. Last but not least, recent data indicate that oviduct EVs may reproduce or even improve the effects of oviduct secretions on embryo quality in pigs (Alcântara-Neto et al., 2022) and cattle (Lopera-Vásquez et al., 2016; Sidrat et al., 2020). Bovine blastocysts exposed to oviduct EVs during early development displayed less cell apoptosis (Sidrat et al., 2020) and significant changes in their phospholipid composition (Banliat et al., 2020a), mitochondrial activity (Sidrat et al., 2020), and gene expression (Bauersachs et al., 2020), which are key factors for further development and conceptus implantation. The underlying mechanisms of EVs probably include direct incorporation of metabolites (Gatien et al., 2019), proteins (Alminana et al., 2017; Banliat et al., 2020b), and microRNAs (Bauersachs et al., 2020) conveyed to the embryonic cells.

## Conclusion and future perspectives

This review shows manyfold evidence for the negative impacts of bypassing the oviduct milieu during *in vitro* embryo production and emphasizes the enormous benefits of mimicking the gamete/embryo–oviductal interactions *in vitro* on sperm selection and embryo development. Altogether, the collected information reveals the unique role of the oviduct, which, to date, cannot be fully replicated *in vitro*. Besides, the current knowledge indicates that additional efforts are needed regarding mimicking the *in vivo* oviduct milieu in ART to overcome the negative impact of the *in vitro* conditions.

To mimic the *in vivo* oviduct milieu *in vitro* is an ambitious and difficult task, since as we discussed here, should cover different events occurring before, during, and after fertilization. Besides, it should consider or involve the various effects of the oviduct epithelium and its secretions on these different processes in gametes and embryos. Finally, new strategies should provide a population of selected sperm with improved ability to fertilize the egg and obtain an embryo of good quality. The most recent *in vitro* models and technologies proposed so far, such as the use of oviduct organoids (Bourdon et al., 2021; Lawson et al., 2023; Gatimel et al., 2025), or a 3D-printing microfluidic “oviduct-on-a-chip” (Ferraz et al., 2018) brought new insights on the oviduct physiology and have demonstrated the ability to enhance sperm survival or motility (Lawson et al., 2023; Gatimel et al., 2025) and embryo quality (Ferraz et al., 2018). However, they are associated with enormous costs and time, and involve difficult experiments, making it very challenging to use in practical applications of ART. Lessons learned from the use of these models or technologies in obtaining gametes and embryos of better quality can be used to develop more feasible models and less cost-effective approaches that can be translated to increased fertilization and pregnancy rates in ART. On the other side, to overcome the impact of the missing oviduct in ARTs, efforts need to be directed to get deeper insights into the plasticity of gametes and embryos to cope with the *in vitro* milieu. Besides, unveiling the fundamental oviductal secretions and components that boost spermatozoa and embryos at the molecular level, will help to develop new *in vitro* supplements for better sperm selection, fertilization, and embryo culture.

## Data Availability

No research data were used.
